# Presentation of Bilateral Facial Paralysis in Melkersson–Rosenthal Syndrome

**DOI:** 10.1155/2021/6646115

**Published:** 2021-01-06

**Authors:** Gustavo Gaitan-Quintero, Loida Camargo-Camargo, Norman López-Velásquez, Miguel González

**Affiliations:** ^1^Neurology Residency Program of Sinú University, Cartagena, Colombia; ^2^Universidad de La Costa, Barranquilla, Colombia; ^3^Duke University, Durham, NC, USA

## Abstract

*Introduction*. Melkersson–Rosenthal syndrome (MRS) is a neuromucocutaneous disorder characterized by the following classic symptom triad: peripheral facial paralysis, orofacial edema, and scrotal or fissured tongue. It is rare, and since most of the patients are oligo- or monosymptomatic, it makes it difficult to diagnose. *Clinical Case*. We present a 26-year-old male patient with a history of sickle cell trait, untreated snoring, and left peripheral facial paralysis when he was 11 years old. This was an overall 20-day clinical profile that started with left peripheral facial paralysis, which was accompanied by moderate-intensity occipital pulsatile headaches. Additionally, the patient experienced paresthesias in the tongue and feelings of labial edema. After one week, he manifested peripheral facial paralysis on the right side. Physical examination revealed bilateral peripheral facial paralysis, mild labial edema, and a scrotal or fissured tongue. The patient received corticosteroids, which resulted in improvement of the edema and facial paralysis. *Discussion*. MRS is a rare disorder that predominantly affects women, typically starting in their 20s or 30s. The etiology is unknown. However, a multifactorial origin that involves environmental factors and a genetic predisposition has been proposed, which causes a dysfunction of the local immune system and autonomic nervous system (ANS) and an appearance of granulomatous inflammation in the lips and tongue. Facial paralysis usually appears later on; however, it can occur from its clinical debut. There are no curative treatments. Therapy is focused on modulating the patient's immune response, and relapses are frequent.

## 1. Introduction

Melkersson–Rosenthal syndrome (MRS) is a neuromucocutaneous disorder without a known etiology. MRS is characterized by a late onset of peripheral facial paralysis with recurring incidences, orofacial edema, and a scrotal or fissured tongue [1]. Even though MRS is rare and has a low clinical presentation of 0.08% in the general population [2], it is estimated that only 8–25% of the patients present the classic symptom triad [3], making diagnosis difficult. Most MRS patients have a monosymptomatic presentation of reoccurring labial edema, which is also predominately found in Miescher syndrome and Miescher's cheilitis granulomatosa [4]. We present a case of MRS in a male patient who came with bilateral facial paralysis and who was also diagnosed with sickle cell trait.

## 2. Clinical Case

**Figure 1:**
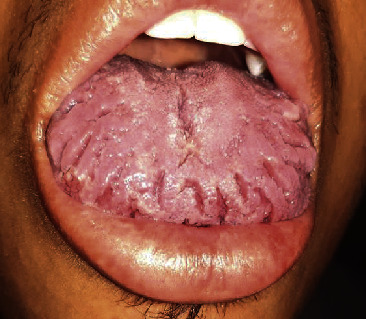
Slight labial edema and a scrotal or fissured tongue.

The patient is a 26-year-old Afro-Colombian male with a history of sickle cell trait and left peripheral facial paralysis from when he was 11 years old. He came for a 20-day clinical profile development that started with feelings of weakness in the right half of the face, causing difficulties in palpebral closure. As a result, the patient experienced moderate-intensity occipital pulsatile headaches that radiated to the mastoid process, feelings of paresthesias in the tongue, and labial edema. The following week, he started to manifest contralateral weakness on one side of his face and the inability for palpebral closure. The physical exam showed evidence of peripheral bilateral facial paralysis, House Brackmann 2 on the left side and 3 on the right side, slight labial edema, and a scrotal or fissured tongue (Figure 1). The blood analysis was normal, expect for sickle cell trait. The cerebral MRI did not present any alternations. The patient was treated with oral prednisolone for 7 days and resulted in complete symptom improvement of his facial paralysis and labial edema.

## 3. Discussion

Melkersson–Rosenthal syndrome (MRS) is a rare disorder described for the first time in 1929 by Ernst Gustaf Melkersson as peripheral facial paralysis and edema in the lips. In 1931, Curt Rosenthal completed the classic symptom triad by adding the presence of a scrotal or fissured tongue [1]. This syndrome predominately affects women in a 2 : 1 ratio, usually starting in their 20s or 30s, and has a range of 1–69 years of age [3, 5]. This clinical case presents a young adult Afro-Colombian male patient. Ethnic distribution for MRS has not been proven. Furthermore, more cases have been reported in the United States and Europe, which could be a misleading statistic according to some authors [6, 7].

The etiology of MRS is unknown. However, a multifactorial origin that involves environmental factors and a genetic predisposition has been proposed, which causes a dysfunction of the local immune and autonomic nervous system (ANS) [1, 8]. Multiple factors have been implicated in its pathogenesis: infectious agents and allergic reactions to a wide array of foods and dietary additives. This includes cobalt, monosodium glutamate, periodontal infections, tonsillitis, adenoidal hypertrophy, orofacial infections due to herpes type 1, and infections from influenza and mycobacteria [1, 5, 7]. There have been reports of familial occurrences that support a genetic etiology and autosomal dominant mutations in genes such as FATP1 (fatty acid transport protein) [9, 10]. The tissue damage from MRS is based on a noncaseating granulomatous inflammation in epithelial cells, Langhans multinucleated giant cells, perivascular lymphocytic infiltrate, and fibrosis [11]. The paralysis has been related to pressure due to edema that passes from the facial canal inside the temporal bone or the granulomatous infiltration from the nerve [1].

The clinical manifestations of MRS vary and are predominately monosymptomatic or oligosymptomatic (two symptoms). According to different case series, only 25–75% of patients with MRS present the classic symptom triad [3, 5]. Facial paralysis is observed in 30–90% of cases and is associated with orofacial edema that occurs in 13–50% of patients [5]. Additionally, facial paralysis is usually the last symptom to manifest, which can occur months or years after the orofacial edema [7]. However, it can develop from its clinical debut, which, almost always, is accompanied with orofacial edema or a fissured tongue [3, 7]. Characteristically, facial paralysis develops within hours or days and is always either partly or completely peripheral. Additionally, the clinical course can be monophasic or recurring, with possibilities of unilateral, bilateral, or alternating facial paralysis. As the clinical profile advances, facial paralysis can become permanent [1]. In this clinical case, the patient presented a bilateral facial paralysis. In a series of cases in China, up to 58% of patients with MRS had bilateral facial paralysis [12]. Peripheral bilateral facial paralysis is most commonly caused by trauma, Guillain–Barré syndrome, Bell's palsy, sarcoidosis, and types of neoplasticism or infectious meningitis [13].

Either in the form of facial edema, labial edema, or both, orofacial edema is present in 70–80% of patients [3, 12], making it the most common monosymptomatic presentation.

When labial edema is recurring, it is most often Miescher syndrome or Miescher's cheilitis granulomatosa [4]. The tongue alteration is present in 30–80% of cases [5] and has been described as scrotal, geographic, fissured, or plicated. It should be taken into account that the tongue alteration is present in 5% of the population, which could make it congenital in 30–80% of patients and of diagnostic value only when accompanied by the other two symptoms [7]. Other clinical neurological manifestations of MRS depend on the degree of inflammation and where structures are compromised near the facial and other cranial nerves. Different symptoms have been described, such as orolingual paresthesias, facial paresthesias, trigeminal neuralgia, weakness and atrophy in the masticatory musculature, dysgeusia, dry eye, hearing loss, vertigo, auricular pain, migraines predominately in the occipital region, lingual weakness and atrophy, anosmia, dysphagia, and diplopia [3, 7, 14–16].

MRS has been associated with other manifestations that are not neurological: lymphadenopathy, conjunctivitis, uveitis, keratitis, blepharitis, and genital lesions [1, 17, 18]. In addition, MRS has shown comorbid associations with psoriasis, tobacco use, obesity, dyslipidemia, hypertension, diabetes mellitus, migraines, gastrointestinal symptoms, psychotic episodes, and obsessive-compulsive disorder [1, 19, 20]. Some complications with the chronification of the clinical profile have been noted, such as sinusitis, candidiasis, facial sun-rash, IgA nephropathy, vitiligo, and abnormalities in thyroid hormones [5].

The diagnosis of MRS is established during clinical suspicion. There do not exist any recognized diagnostic criteria nor a definitive clinical scoring guide. Some authors suggest that the presence of the classic symptom triad of recurring peripheral facial paralysis, orofacial edema, and a scrotal or fissured tongue is sufficient for a diagnosis [2, 7]. Also, it has been proposed that there must be a minimum of two symptoms in order to establish a clinical diagnosis, requiring a biopsy in monosymptomatic cases such as with Miescher cheilitis [4, 5]. A lip biopsy helps reinforce the diagnosis by confirming a noncaseating granulomatous inflammation [2, 11]. Other differential diagnoses are primarily related to granulomatous disorders, such as Crohn's disease and sarcoidosis, that can overlap with many of the classic triad of symptoms. Furthermore, diagnoses to consider that can imitate mono- or oligosymptomatic presentations are reactions to antibodies, recurring Bell paralysis, Wegener's vasculitis, amyloidosis, and infectious agents [21]. Complimentary exams should be conducted when the clinical profile is confusing, the patient's age is unusual in relation to the start of their symptoms, they have an autoimmune history, or a lymphoproliferative disease [2].

Some exams that we recommend are glucose readings, blood count, globular sedimentation rate, bran scan, brain MRI, thorax X-ray, serum levels of the angiotensin-converting enzyme, antibodies ANA and ANCA, and thyroid tests. Other exams that are more specific to the clinical profile are a colonoscopy, HLA for inflammatory bowel syndrome, C1 inhibitor, and genetic tests [5]. Also, electrodiagnostic tests, such as auditory evoked potentials, blink reflex, electromyography, and neuroconduction of the facial nerve, can complement the study. Some findings describe a global or partial rate loss of the facial nerve branches, an increase in distal latency, and a decrease of compound muscular action potentials, which correlates with entrapment neuropathy [22].

In the current case, immunological exams performed were negative. The only exam that resulted in an abnormal reading was the sickle cell trait. In the literature, there has not been a demonstrated association between MRS, sickle cell anemia, and sickle cell trait. A biopsy of the lip was not performed due to the presence of the classic clinical *t* associated to the characteristic clinical profile.

There does not exist a curative treatment. Pharmacological therapies are focused on modulating the immune response, making corticoids the mainstay treatment. There are not any clinical trials that suggest a type of corticoid or a dosage. Using corticoid therapy has been shown to cause a 50–80% improvement in patients. The treatment plan most utilized is a full dosage of oral corticoids for a week and a gradual reduction in the second week. In the most severe cases, intravenous methylprednisolone is given [5].

In cases of persistent orofacial edema, some authors suggest the use of intralesional corticoids, such as betamethasone and triamcinolone. Additionally, antibiotics with anti-inflammatory properties, such as doxycycline and minocycline, have been used as an adjuvant therapy [23, 24]. The main indicator to implement immunosuppressors is the comorbidity of an autoimmune system disease. Some case series have shown benefits of using monoclonal antibodies adalimumab and infliximab [5]. Other alternatives used are dietary changes, avoiding possible allergenics and nutraceuticals supplements [25]. Other medications such as anti-inflammatories, steroids, and antihistamines have been utilized to improve symptoms [26]. The effectivity of the pharmacological therapies is moderate and relapses occur frequently. The prognosis for permanent damage to the facial nerve when there is recurring paralysis is poor in the majority of the cases and is even worse in patients with recurring Bell paralysis [27]. Surgical treatment is reserved for patients with recurring facial paralysis and consists in the decompression of the facial nerve by means of a mastoidectomy. One study showed that none of the patients presented a recurrence of facial paralysis and about 90% of the patients recovered to an almost normal functioning level [28].

## 4. Conclusions

MRS is an infrequent disorder that is characterized by the classic symptom triad of orofacial edema, facial paralysis, and a fissured tongue but more commonly is mono- or oligosymptomatic. In the absence of other clinical information that points to another diagnosis, it is suggested that a clinical diagnosis can be established when two or three symptoms of the classic symptom triad are present. Occasionally, bilateral facial paralysis can initially manifest and should be considered in the broad range of the differential diagnosis.

## Data Availability

Data used to support the findings of this study can be obtained from the corresponding author upon request.

## References

[1] Basman A., Gumusok M., Degerli S., Kaya M., Toraman Alkurt M. (2017). Melkersson-rosenthal syndrome: a case report. *Journal of Istanbul University Faculty of Dentistry*.

[2] Cancian M., Giovannini S., Angelini A. (2019). Melkersson–Rosenthal syndrome: a case report of a rare disease with overlapping features. *Allergy, Asthma & Clinical Immunology*.

[3] Feng S., Yin J., Li J., Song Z., Zhao G. (2014). Melkersson-Rosenthal syndrome: a retrospective study of 44 patients. *Acta Oto-Laryngologica*.

[4] Critchlow W. A., Chang D. (2014). Cheilitis granulomatosa: a review. *Head and Neck Pathology*.

[5] Dhawan S. R., Saini A. G., Singhi P. D. (2020). Management strategies of Melkersson-rosenthal syndrome. *A Review*.

[6] Aydın S., Öztürk S., Faraşoğlu A., Çakıl T., Çoruk S. J. S. (2018). Melkersson–Rosenthal syndrome. *A Case Report*.

[7] Ozgursoy O. B., Karatayli Ozgursoy S., Tulunay O., Kemal O., Akyol A., Dursun G. (2009). Melkersson-Rosenthal syndrome revisited as a misdiagnosed disease. *American Journal of Otolaryngology*.

[8] Brożek-Mądry E., Jaremek-Ochniak W., Straburzyński M., Krzeski A. (2019). Lower lip Oedema-Melkersson-Rosenthal Syndrome or Cheilitis Granulomatosa. *New American Journal of Otolaryngology Research*.

[9] Xu X., Guan L., Lv Y. (2017). Exome sequencing identifies FATP1 mutation in Melkersson-Rosenthal syndrome.

[10] Pei Y., Beaman G. M., Mansfield D., Clayton-Smith J., Stewart M., Newman W. G. (2019). Clinical and genetic heterogeneity in Melkersson-rosenthal syndrome. *European Journal of Medical Genetics*.

[11] Bohra S., Kariya P. B., Bargale S. D., Kiran S. (2015). Clinicopathological significance of Melkersson-Rosenthal syndrome. *BMJ Case Reports*.

[12] Tang J.-J., Shen X., Xiao J.-J., Wang X.-P. (2016). Retrospective analysis of 69 patients with Melkersson-Rosenthal syndrome in mainland China.

[13] Gaudin R. A., Jowett N., Banks C. A., Knox C. J., Hadlock T. A. (2016). Bilateral facial paralysis: a 13-year experience. *Plastic and Reconstructive Surgery*.

[14] Zeng W., Geng S., Niu X., Yuan J. (2010). Complete Melkersson–Rosenthal syndrome with multiple cranial nerve palsies.

[15] Aluclu M. U., Keklikci U., Guzel A., Unlu K., Tatli M. (2008). Melkersson-Rosenthal syndrome with partial oculomotor nerve palsy. *Annals of Saudi Medicine*.

[16] Liu R., Yu S. (2013). Melkersson-Rosenthal syndrome: a review of seven patients. *Journal of Clinical Neuroscience*.

[17] Chu Z., Liu Y., Zhang H., Zeng W., Geng S. (2016). Melkersson-Rosenthal syndrome with genitalia involved in a 12-year-old boy. *Annals of Dermatology*.

[18] Ogawa T., Sotto M. N., Hoang M. P., Hoang M. P., Selim M. A. (2020). Granulomatous dermatitis and others. *Hospital-Based Dermatopathology: An Illustrated Diagnostic Guide*.

[19] Shalom G., Bodner L., Halevy S. (2019). Melkersson-Rosenthal Syndrome: the possible role of comorbidities in the etiopathogenesis. Giornale italiano di dermatologia e venereologia: organo ufficiale, Societa italiana di dermatologia e sifilografia.

[20] Kayhan F., İlik F., Kayhan A. (2015). Obsessive–compulsive disorder concurrent with Melkersson-Rosenthal Syndrome: a case report. *General Hospital Psychiatry*.

[21] Elias M. K., Mateen F. J., Weiler C. R. (2013). The Melkersson–Rosenthal syndrome: a retrospective study of biopsied cases. *Journal of Neurology*.

[22] Saini A. G., Sankhyan N., Padmanabh H., Das A., Singhi P. (2016). Recurrent facial palsy and electrophysiological findings in oligosymptomatic Melkersson rosenthal syndrome. *The Indian Journal of Pediatrics*.

[23] Oudrhiri L., Chiheb S., Marnissi F., Zamiati S., Benchikhi H. (2012). Successful treatment of Miescherʼs cheilitis in Melkersson-Rosenthal syndrome with betamethasone injections and doxycycline.

[24] Bacci C., Valente M. L. (2010). Successful treatment of cheilitis granulomatosa with intralesional injection of triamcinolone.

[25] Espinoza I., Navarrete J., Benedetto J., Borzutzky A., Roessler P., Ortega-Pinto A. J. A. (2018). Orofacial granulomatosis and diet therapy: a review of the literature and two clinical cases.

[26] Rašković S., Bolpačić J., Perić-Popadić A., Jovicić Ž., Mišković R., Bogić M. (2015). Clinical characteristics and treatment of Melkersson-Rosenthal syndrome: overview of six patients.

[27] Wang J., Li P., Jin X., Xu Y., Zhang X. J. (2015). Outcomes of recurrent facial palsy in Melkersson Rosenthal syndrome. *Annals of Otology, Rhinology & Laryngology*.

[28] Tan Z., Zhang Y., Chen W., Gong W., Zhao J., Xu X. (2015). Recurrent facial palsy in Melkersson Rosenthal syndrome: total facial nerve decompression is effective to prevent further recurrence. *American Journal of Otolaryngology*.

